# SABCS 2020: update on triple-negative and metastatic HER2-positive breast cancer

**DOI:** 10.1007/s12254-021-00722-4

**Published:** 2021-06-29

**Authors:** Rupert Bartsch

**Affiliations:** Department of Medicine 1, Division of Oncology, Medical University of Vienna, Waehringer Guertel 18–20, 1090 Vienna, Austria

**Keywords:** San Antonio Breast Cancer Symposium 2020, Metastatic HER-positive breast cancer, Triple-negative breast cancer, Highlights, Review, Conference report

## Abstract

One year into the severe acute respiratory syndrome coronavirus type 2 (SARS-CoV-2) pandemic, the 2020 San Antonio Breast Cancer Symposium (SABCS) was another large congress held in a virtual format. Despite these circumstances, clinically relevant data were presented, and this short review focuses on developments in the fields of triple-negative breast cancer (TNBC) and metastatic HER2-positive breast cancer. A quality-of-life (QoL) analysis from IMPassion031 showed that adding atezolizumab to neoadjuvant chemotherapy was not associated with a detrimental effect on QoL, while the burden of treatment-induced side effects increased with each cycle of neoadjuvant therapy in both treatment arms. KEYNOTE-355 evaluated the addition of pembrolizumab to chemotherapy as first-line treatment in metastatic TNBC (mTNBC); a significant improvement of progression-free survival (PFS) was reported in the pembrolizumab arm. At the 2020 SABCS, results with respect to different chemotherapy backbones were reported and the benefit of pembrolizumab was maintained irrespective of the type of taxane. Disappointingly, the phase III IPATunity130 study could not confirm a PFS improvement with the AKT inhibitor ipatasertib when added to paclitaxel as first-line treatment in mTNBC. A biomarker analysis from the phase III ASCENT study showed that the antibody–drug conjugate sacituzumab govitecan was superior to chemotherapy by investigator’s choice independent of Trop‑2 expression and *BRCA* mutation status. In HER2-positive breast cancer, the PRECIOUS trial suggested a small albeit significant benefit with reinduction of pertuzumab in later treatment lines in patients progressing on prior dual HER2-blockade in the first- or second-line setting. The HER2-specific tyrosine kinase inhibitor tucatinib when added to trastuzumab and capecitabine was shown to improve PFS and overall survival (OS) over trastuzumab and capecitabine alone in pretreated patients in the randomized HER2CLIMB trial; this benefit was apparently independent of hormone-receptor expression. An update from the DESTINY-Breast01 trial reported a median PFS of 19.4 months with trastuzumab deruxtecan in heavily pretreated patients. Finally, an analysis from the PERTAIN trial with > 6 years median follow-up showed excellent OS in patients with luminal B/HER2-positive receiving first-line trastuzumab/pertuzumab in combination with endocrine therapy suggesting that chemotherapy-free treatment is an option in highly selected patients.

## Introduction

Due to the ongoing severe acute respiratory syndrome coronavirus type 2 (SARS-CoV-2) pandemic, the 2020 San Antonio Breast Cancer Symposium (SABCS) was another meeting held only in a virtual format. Despite this drawback, interesting results were presented, and this short review focuses on clinically relevant data in the field of triple-negative breast cancer (TNBC) and metastatic HER2-positive breast cancer.

## Triple-negative breast cancer

### Immunotherapy

Defining the role of immunotherapy with immune checkpoint inhibitors remains a major scientific focus in TNBC. In the neoadjuvant setting, addition of the PD-L1 inhibitor atezolizumab to neoadjuvant chemotherapy yielded an increase of pathologic complete remission rate of 16.5% in the prospective randomized phase III IMPassion031 trial [1]. At the 2020 SABCS, quality-of-life (QoL) data were presented [2]. In the atezolizumab group, no additional reduction of overall health-related QoL or specific treatment-related symptoms such as fatigue, nausea/vomiting, or diarrhoea were reported. In both arms, an increase of side-effect burden was observed with each cycle during the neoadjuvant phase of the trial. While these data are reassuring with regard to the use of immunotherapy, they also show that chemotherapy-induced side effects remain a major burden.

Several studies investigated the role of checkpoint inhibitors in metastatic TNBC (mTNBC) either alone or in combination with conventional chemotherapy. IMPassion130 established the combination of atezolizumab and nab-paclitaxel as a standard approach in the first-line treatment of patients with PD-L1-positive (immune cell score) mTNBC [3]. In contrast, no benefit in terms of progression-free survival (PFS) or overall survival (OS) was observed when atezolizumab was combined with solvent-based paclitaxel in the IMPassion131 trial [4]. The reason for this difference, while often attributed to the need for corticosteroid comedication with conventional paclitaxel, remains largely unresolved. In KEYNOTE-355, the addition of the PD‑1 inhibitor pembrolizumab to three different chemotherapy backbones (paclitaxel, nab-paclitaxel, carboplatin/gemcitabine) was evaluated; initial results have already been published and the addition of pembrolizumab yielded a clinically meaningful prolongation of PFS from 5.6 to 9.7 months (hazard ratio [HR] 0.65) in patients with PD-L1-positive (combined positive score ≥ 10) mTNBC [5]. In the light of the aforementioned contradicting outcomes in the IMPassion trials, the effect of pembrolizumab in the two taxane groups was eagerly awaited. While no stratification for the type of taxane was performed, results of KEYNOTE-355 suggest no difference between the two taxane groups (PFS nab-paclitaxel 5.5 vs. 9.9 months, HR 0.57; PFS solvent-based paclitaxel 3.6 vs. 9.6 months, HR 0.33) [6]. Approval by the European Medicines Agency (EMA) is awaited once OS data are available.

### IPATunity130

In the randomized phase II LOTUS trial, addition of the AKT inhibitor ipatasertib to first-line paclitaxel improved PFS over paclitaxel alone in treatment naïve mTNBC patients [7]; the benefit was more pronounced in patients with tumours harbouring alterations in the PI3K/AKT pathway, suggesting a clinically significant role of AKT inhibitors in this specific patient population. Therefore, cohort A of the phase III IPATunity130 trial randomized 255 patients with mTNBC and PI3K/AKT/PTEN alterations 2:1 to first-line paclitaxel with ipatasertib or placebo [8]. At a median follow-up of 8.3 months, no difference in terms of PFS or response rate was observed between the groups. No differential outcomes were observed in patients with activating *PIK3CA*/*AKT1* mutations or PTEN alterations without activating mutations as well. While these data are clearly disappointing, they once again show that despite growing biological understanding, phase III trials are still required.

### ASCENT

Sacituzumab govitecan (SG) is an antibody–drug conjugate (ADC) consisting of SN-38, the active metabolite of irinotecan, and a humanized Trop‑2 antibody, an epithelial antigen commonly expressed in TNBC. The ASCENT trial randomized patients with pretreated mTNBC to SG or chemotherapy by physician’s choice. Results of this phase III study have already been presented, and SG yielded a significant and clinically relevant improvement of PFS and OS [9]. In a biomarker analysis presented at the 2020 SABCS, it was shown that the benefit in favour of SG was maintained independent of the degree of Trop‑2 expression, although numerically the smallest PFS and OS difference was observed in patients with Trop‑2 low-expressing tumours (PFS: Trop‑2 high 6.9 vs. 2.5 months, Trop‑2 medium 5.6 vs. 2.2 months, Trop‑2 low 2.7 vs. 1.6 months; OS: Trop‑2 high 14.2 vs. 6.9 months, Trop‑2 medium 14.9 vs. 6.9 months, Trop‑2 low 9.3 vs. 7.6 months) [10]. Regarding germline *BRCA1/2* mutations, superiority of SG was maintained independent of mutation status. In summary, these data suggest that SG is an important addition to the therapeutic options in TNBC once EMA approval is obtained.

In the prespecified subgroup of patients with stable brain metastases (BM) at baseline (*n* = 61), however, SG was not superior to conventional chemotherapy in terms of PFS and OS [11]. As patients with active brain metastases (i.e., newly diagnosed or progressing after prior radiotherapy) were excluded, a final conclusion regarding the activity (or lack thereof) of SG in BM cannot be drawn. Indeed, in a pilot study accruing patients with glioblastoma multiforme and breast cancer BM, responses were observed in 2/6 patients with measurable breast cancer BM according to RANO-BM (Response Assessment in Neuro-Oncology Brain Metastases) criteria; 7/10 breast cancer patients remained progression-free for at least 6 months [12]. Based upon these results, the single-arm phase II SWOG S2007 trial investigating SG in patients with active HER2-negative breast cancer BM is currently ongoing (NCT04647916).

## Metastatic HER-positive breast cancer

### PRECIOUS

Treatment in multiple lines—the continuation of trastuzumab with alternating chemotherapy combination partners at each progression—is a well-established concept in metastatic HER2-positive breast cancer. In contrast, the potential benefit of reinducing pertuzumab after progression on first-line trastuzumab/pertuzumab is ill defined. The phase III PRECIOUS trial randomized 219 patients with HER2-positive metastatic breast cancer (mBC) after progression on trastuzumab/pertuzumab/chemotherapy as first- or second-line therapy and one subsequent treatment line (including T-DM1) to chemotherapy plus trastuzumab or chemotherapy plus trastuzumab and pertuzumab in the third- or fourth-line settings [13]. Reinducing dual HER2-inhibition resulted in a statistically significant albeit numerically small PFS improvement from 4.2 to 5.3 months (HR 0.76; *p* = 0.0217); a numerical improvement in OS was observed as well. While these results are interesting, they appear somewhat outdated in the presence of the third-generation HER2-directed drugs tucatinib and trastuzumab deruxtecan (T-DXd).

### Update on HER2CLIMB and DESTINY-Breast01

The HER2-specific tyrosine kinase inhibitor (TKI) tucatinib and the ADC T‑DXd have clinically relevant activity in pretreated HER2-positive breast cancer and both drugs were recently approved by EMA.

In the large phase II HER2CLIMB study, patients were randomized to tucatinib or placebo in combination with trastuzumab and capecitabine. All patients had received prior therapy with trastuzumab, pertuzumab and T‑DM1 and addition of tucatinib yielded a clinically relevant and statistically significant improvement of PFS and OS [14]. Of note, a large fraction of patients had active BM at baseline and the effect of tucatinib was particularly pronounced in this subset [15]. At the 2020 SABCS, a comparison of outcomes in dependence upon oestrogen-receptor (ER) expression was presented [16]. While OS curves suggest a larger benefit in the non-luminal/HER2-positive (i.e. ER-negative) subset, PFS hazard ratios were comparable (HR [ER-negative] 0.54; HR [ER-positive] 0.58). Therefore, these data suggest a benefit of tucatinib irrespective of ER expression and OS curves may separate later in ER-positive disease due to the specific biology of luminal B/HER2-positive breast cancer.

In this single-arm phase II DESTINY-Breast01 trial, T‑DXd generated a response rate of 60.9% in a heavily pretreated population with a median of six prior treatment lines [17]. An update with a median follow-up of 20.5 months reported a median PFS of 19.4 months (95% confidence interval [CI] 14.1-NE [not established]); median OS was estimated at 24.6 months, but results are still immature [18]. With the caveat of the single-arm phase II design, updated data therefore confirm the clinically relevant activity of T‑DXd. Interstitial lung disease (ILD) was already reported as an adverse event of special interest; from the first to the second analysis (August 2019 and June 2020, respectively), the ILD rate showed a slight increase from 13.8% to 15.2% with one additional death from ILD reported. Therefore, ILD appears not correlated with the cumulative T‑DXd dose but careful monitoring and early intervention is required.

### PERTAIN

In luminal B/HER2-positive mBC, several studies investigated the chemotherapy-free addition of HER2-directed drugs to endocrine therapy. While this strategy was shown to improve PFS over endocrine treatment alone and has a favourable toxicity profile, no OS benefit was observed to date. The randomized phase II PERTAIN trial asked the question whether patients benefitted from the addition of pertuzumab to first-line trastuzumab in combination with an aromatase inhibitor (AI). Induction chemotherapy (docetaxel or paclitaxel) was allowed by investigators choice. Results have already been published, and addition of pertuzumab improved PFS from 15.8 to 18.9 months (HR 0.65; 95% CI 0.48–0.89; *p* = 0.007) [19]. A final study analysis with a median follow-up of more than 6 years was presented at the 2020 SABCS [20]. Here, an OS in excess of 5 years was reported in the group of patients with first-line dual HER2 inhibition plus AI without induction chemotherapy, suggesting that chemotherapy-free treatment may be reasonably possible in a selected subset of low-risk patients or patients who are not deemed candidates for more intense (standard) therapy.

#### Take home message

In the field of immunotherapy, the quality of life (QoL) analysis from the IMpassion031 trial suggests that addition of atezolizumab to neoadjuvant chemotherapy has no detrimental effect on QoL; in KENOTE-355, addition of pembrolizumab to first-line chemotherapy in PD-L1-positive mTNBC improved progression-free survival (PFS) irrespective of the taxane backbone; in the phase III IPATunity130 trial, the AKT inhibitor ipatasertib yielded no PFS benefit when added to first-line paclitaxel in metastatic TNBC with alterations in the PI3K/AKT pathway; a biomarker analysis from the ASCENT trial confirmed the superiority of sacituzumab govitecan over conventional chemotherapy in pretreated mTNBC patients irrespective of Trop‑2 expression or *BRCA* mutation status.

Updated results from the HER2CLIMB and DESTINY-Breast01 trials confirmed the high activity of the third-generation HER2-directed drugs tucatinib and T‑DXd in pretreated metastatic breast cancer (mBC) patients. The PFS benefit seen with the addition of tucatinib to trastuzumab plus capecitabine was irrespective of hormone-receptor expression, while the overall survival (OS) curves seem to separate later in luminal B/HER2-positive BC. With longer follow-up, estimated median PFS with T‑DXd in the single-arm DESTINY-Breast01 study was 19.4 months. Finally, the end-of-study analysis of the PERTAIN trial reported excellent long-term outcomes with first-line endocrine therapy plus trastuzumab and pertuzumab in luminal B/HER2-positive patients, suggesting that chemotherapy-free first-line treatment may be considered in a selected population.
